# Effects of Exercise during Pregnancy on Postpartum Depression: A Systematic Review of Meta-Analyses

**DOI:** 10.3390/biology10121331

**Published:** 2021-12-15

**Authors:** Priscila Marconcin, Miguel Peralta, Élvio R. Gouveia, Gerson Ferrari, Eliana Carraça, Andreas Ihle, Adilson Marques

**Affiliations:** 1 Faculdade de Motricidade Humana, Universidade de Lisboa, 1499-002 Cruz Quebrada, Portugal; priscilamarconcin@fmh.ulisboa.pt; 2 Centro Interdisciplinar de Estudo da Performance Humana (CIPER), Faculdade de Motricidade Humana, Universidade de Lisboa, 1499-002 Cruz Quebrada, Portugal; mperalta@fmh.ulisboa.pt; 3 Instituto de Saúde Ambiental (ISAMB), Universidade de Lisboa, 1649-004 Lisbon, Portugal; 4 Departamento de Educação Física e Desporto, Universidade da Madeira, 9000-390 Funchal, Portugal; erubiog@staff.uma.pt; 5 Interactive Technologies Institute, LARSyS, 9000-390 Funchal, Portugal; 6 Escuela de Ciencias de la Actividad Física, el Deporte y la Salud, Universidad de Santiago de Chile (USACH), Santiago 7500618, Chile; gerson.demoraes@usach.cl; 7 Centro de Investigação em Desporto, Educação Física, Exercício e Saúd (CIDEFES), Faculdade de Educação Física e Desporto, Universidade Lusófona de Humanidades e Tecnologias, 1749-024 Lisbon, Portugal; elianacarraca@gmail.com; 8 Center for the Interdisciplinary Study of Gerontology and Vulnerability, University of Geneva, 1205 Geneva, Switzerland; andreas.ihle@unige.ch; 9 Swiss National Centre of Competence in Research LIVES—Overcoming Vulnerability: Life Course Perspectives, 1015 Lausanne, Switzerland; 10 Department of Psychology, University of Geneva, 1205 Geneva, Switzerland

**Keywords:** mental health, physical activity, sports

## Abstract

**Simple Summary:**

Postpartum depression (PPD) is a public health problem. Exercise is a nonpharmacologic alternative to deal with PPD. This study conducted a systematic review of previous meta-analyses and an exploratory pooled analysis regarding the effects of exercise on depressive symptoms among women during the postpartum period. We searched for previous meta-analyses of experimental studies. Of the 52 records selected, we included five in the analyses, because they were focused on PPD. From the results, it was clear that exercise had a significant but small effect on depressive symptoms. This study shows that exercise is effective in reducing PPD symptoms.

**Abstract:**

Postpartum depression (PPD) is a public health issue. Exercise is a nonpharmacologic alternative to deal with PPD. This study conducted a systematic review of previous meta-analyses and an exploratory pooled analysis regarding the effects of exercise on depressive symptoms among women during the postpartum period. We searched for previous meta-analyses of randomised controlled trials on PubMed, Web of Science and Scopus, date of inception to 31 May 2021. The methodological quality was assessed using the Assessment of Multiple Systematic Reviews 2 (AMSTAR2) instrument. We pooled the standardised mean differences from the selected studies. Of the 52 records screened, five were included. The results revealed a significant moderate effect of exercise on depressive symptoms among women during the postpartum period (SMD = −0.53; 95% CI: −0.80 to −0.27, *p* < 0.001). The pooled effect of the five meta-analyses established that exercise had a significant, small effect on depressive symptoms (SMD = −0.41; 95% CI: −0.50 to −0.32, *p* < 0.001). Our study indicates that exercise is effective in reducing PPD symptoms. Compared with traditional control approaches (psychosocial and psychological interventions), exercise seems have a superior effect on PPD symptoms. The implications of the present synthesis of past meta-analytical findings to guide health policies and research are discussed.

## 1. Introduction

The prevalence of postpartum depression (PPD) varies between 11.9% and 19.2% during the perinatal period [1,2]. PPD refers to minor and major depression incidents that occur during pregnancy or shortly after (up until 12 months after birth) [3]. Due to its similarities to pregnancy and puerperium discomforts symptoms, PPD frequently goes undetected [4]. The symptoms of PPD embrace feeling sad or having a depressed mood, being uninterested in the new-born, unreasonable crying and fear of injuring or harming the baby [5]. Consequently, PPD can negatively impact the mother’s well-being and the baby’s development [6]. The impact on a child can be short for cognitive and motor development [7]. It can also be expressed in the long term, namely on psychological outcomes during adolescence [8]. For women, the impact can also be in the long term, as those reporting thoughts of self-harm after giving birth are known to have an increased risk of morbidity for the next seven years [9]. In addition, frequently, women with PPD also experience anxiety disorders [10]. Regarding risk factors, a history of major depression, lifetime anxiety disorder diagnosis and adverse life events were all considered important predictors of PPD [11].

PPD can be managed with psychotherapy, medication, lifestyle changes, a supportive environment or a combination of these [12]. Although medication is a feasible alternative, many women have constraints due to continuing breastfeeding [13]. Therefore, exercise can be an alternative that could help to deal with PPD. Exercise can be used as a preventive or treatment of mild depression at an early stage and as an addition to a treatment plan for major depressive disorder [12]. Exercising during pregnancy and postpartum improves psychological health and also benefits physical fitness [14,15], weight gain control [16,17] and the prevention or reduction of musculoskeletal discomfort and pain [18,19]. Therefore, the American College of Obstetrics and Gynaecologists has recommended that women during pregnancy and postpartum engage in moderate-intensity physical activity almost every day for 30 min a day [20].

Several meta-analyses have examined the exercise interventions’ efficacy in preventing or reducing PPD symptoms [21,22,23]. These meta-analyses vary in the aim, the type of exercise intervention and study quality, hampering the overarching conclusions. However, no study has been done to synthesise these findings. Therefore, an overview of the existing systematic reviews is an efficient way to gather and summarise the best available evidence on the effectiveness of an intervention [24]. Additionally, it can provide useful and update evidence for practitioners and clinicians. Thus, this systematic review and meta-analysis aimed to (1) appraise past meta-analyses regarding the effects of exercise on PPD and (2) synthesise past meta-analytical findings to guide health policies and research.

## 2. Materials and Methods

### 2.1. Literature Search

This systematic review protocol was registered at PROSPERO (CRD42021254814), and the systematic review itself followed the PRISMA 2020 guidelines [25].

For selecting manuscripts, first, a screening based on titles and abstracts was performed, followed by full-text reads to establish the final selection. In both the screening and full-text read stages, two researchers performed the analysis (PM and AM). In case of disagreement, another researcher (MP) was asked to mediate.

We conducted a broad search on meta-analyses published until 31 May 2021 using PubMed, Web of Science and Scopus. The search terms and strategy were: “physical activ*” OR “physical inactiv” OR exercise OR training OR sport* OR fitness OR “movement behavio*” OR walking OR running OR yoga OR jogging OR swimming OR cycl* AND depress* OR “mental health” OR mood OR “psychological health” OR “psychological function*” OR “mental function*” OR worries OR worry OR “depressive disorder*” OR “baby blues” AND postpartum OR postpartum OR postnatal OR post-natal NOT Rats. They were limited to English articles.

### 2.2. Eligibility Criteria

The included articles met the following PICOS (participants, intervention, comparison, outcome and study design) guidelines [26]: (1) Population: women during the first year postpartum, (2) Intervention: any exercise intervention, (3) Comparison: any comparison condition, (4) Outcome: PPD or depressive symptoms and (5) Study design: meta-analyses of RCTs published from data inception to 31 May 2021. Meta-analyses involving animals were excluded.

### 2.3. Quality Assessment

The methodological quality of the studies was assessed by two independent authors (PM and AM) using the Assessment of Multiple Systematic Reviews (AMSTAR). This instrument uses dichotomous scoring (0 or 1) for assessing systematic reviews and meta-analyses’ rigour. The scores range from 0 to 11, and studies are graded as high-quality (score between 8 and 11), medium-quality (score between 4 and 7) and low-quality (score between 0 and 3) [27]. The authors discussed the discrepancies in grading and reached a consensus.

### 2.4. Data Extraction

The study characteristics were extracted based on PICO criteria (population, intervention, comparison and outcomes) [26] by one author, as well as standardised mean differences (SMD) from meta-analytic comparisons. The SMD was classified as trivial if <0.20, small between 0.20 and 0.49, medium between 0.50 and 0.79 and large if >0.80 [28]. The following information was extracted: number of randomised controlled trials (RCTs) and sample included in each comparison.

### 2.5. Statistical Analysis

Heterogeneity data of the meta-analytic comparisons was assessed using the I^2^ statistics, where reported values of 0–25%, 2550%, 50–75% and >75% indicated, respectively, low, moderate, large and very large inconsistencies [29]. Fixed effects were used in the meta-analysis of the meta-analyses. Pooled effect sizes were expressed by the standardised mean differences (SMD) of the effects of exercise on postpartum depressive symptoms. In addition, random effects were used to obtain the pooled effect size (i.e., SMD) derived from the RCTs included in the identified meta-analysis studies, excluding the overlapped studies/RCTs. The heterogeneity using the I^2^ statistic, tau-square and Z-test for the overall effect were assessed. All statistical analyses and calculations were performed by Comprehensive Meta-Analysis version 3.0 software. In cases of information unavailability in the study, the authors were contacted and asked to complement the data extraction.

## 3. Results

### 3.1. Included Meta-Analyses

**Figure 1:**
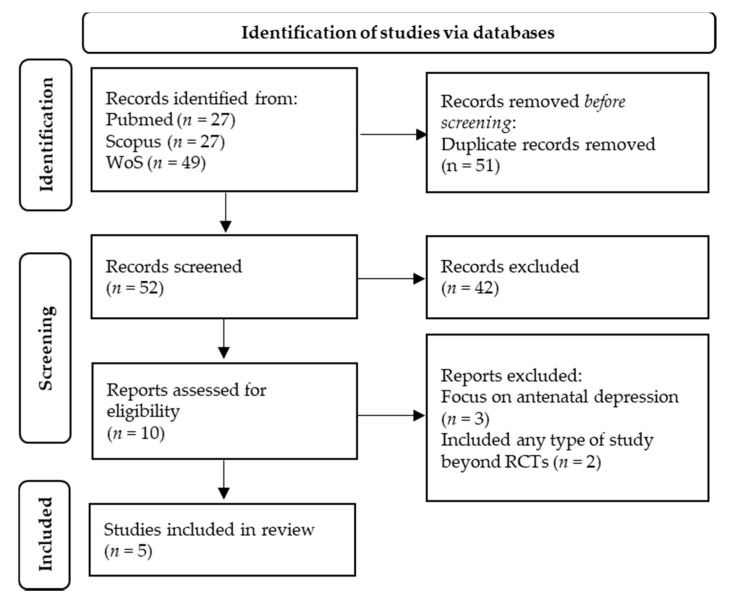
Preferred Reporting Items for Systematic Reviews and Meta-analysis (PRISMA) flow diagram of the search and selection process for the meta-analyses included in the current study.

Figure 1 presents the PRISMA flow diagram of the selection process. The database search included 103 publications. After removing duplicates, 52 articles remained. During screening (title and abstract), 42 articles were excluded. Some interventions did not consist of exercise or physical activity; others did not include women in the postpartum period, and some were not meta-analyses. The remaining 10 articles were designated for a full-text read. Five articles were excluded, because the focus was on antenatal depression, including any study beyond RCTs. Finally, five meta-analyses were included in the present study [30,31,32,33,34].

### 3.2. Characteristics of Meta-Analyses

Table 1 presents a summary of the included meta-analyses following the PICO search strategy. All five included meta-analyses reported the effects of exercise interventions on postpartum depressive symptoms.

#### 3.2.1. Population

In total, 63 RCTs involving 6141 participants were included. The eligibility criteria for included participants varied between studies, including:

(a) primiparous or multiparous postnatal women [30].
(b) women who were between 4 weeks and 18 months postpartum [35].
(c) postpartum women with and without depression [32].
(d) women up to 1 year postpartum [33].
(e) pregnant women with a single foetus and an uncomplicated pregnancy, or women who had a child aged between 6 weeks and 18 months [34].

It excluded pregnant women and women with psychiatric diagnoses other than depression.

#### 3.2.2. Intervention

Regarding exercise interventions, the studies included different types of interventions: coaching-based, motivational and counselling interventions [30,33]; supervised, planned exercise [30,32,35]; home-based program [34]; aerobic exercise [33,34]; stretching and breathing exercises; a walking program; mixed cardiovascular and strength exercises and Pilates and yoga [34]. Regarding the duration of the intervention, in the Carter study [30], 76% continued up to 12 weeks, and the duration of the supervised delivered sessions ranged from 30 to 90 min. Most sessions were delivered at a moderate intensity, and the frequency ranged from 1 to 4. In the Daley study [31], all trials included interventions of 12 weeks, and the duration of the sessions ranged from 30 to 45 min. In the McCurdy study [32], the frequency of the exercise ranged from one to five times per week for 30–60 min and lasted between 6 weeks and 12 months. In the Pritchett study [33], the interventions ranged from 4 weeks to 6 months in duration, and most of the included studies aimed to achieve 30 min of moderate activity three to five times weekly. In the León study [34], the session frequency varied from 1 to 5 days per week, and the intensity was measured as low, moderate and moderate to high.

#### 3.2.3. Comparison

Exercising was compared to any control condition (including exercise) [30], usual care [32,33,35], social support intervention [35] and any intervention during pregnancy and the postpartum period [34].

#### 3.2.4. Outcomes Measure

The main outcome measure across the studies was depressive symptoms, using the Edinburgh Postnatal Depression Scale (EPDS) [30,32,34,35], Patient Health Questionnaire [30], Beck Depression Inventory [34,35], clinical diagnoses [33,35], Center for Epidemiological Studies Depression Scale (CES-D) and Hamilton Depression Rating Scale (HDRS) [32].

#### 3.2.5. Adverse Events

Little information was disclosed about potential adverse events. Poyatos-Leon et al. [34] mentioned that most studies did not disclose adverse effects attributable to interventions. The other four meta-analyses did not report information about adverse events from the included studies.

### 3.3. Methodological Quality

Table 2 presents an item-by-item quality assessment for each study using the AMSTAR 2 instrument. The meta-analyses by Carter et al. [30], Pritchett et al. [33] and Poyatos-León et al. [34] were classified as moderate quality, and Daley et al. [35] and McCurdy et al. [32] were classified as low quality essentially, because both studies did not discuss how the publication biases impacted the results of their reviews.

### 3.4. Quality of Evidence

Only Carter’s study [30] reported a quality of evidence and presented that the overall quality of evidence for exercise on depression symptoms was low and with a small treatment effect.

### 3.5. Results of Individual Meta-Analyses

The review findings of the individual meta-analysis are summarised in Table 3. All five meta-analyses reported the effects of different types of exercise interventions (e.g., coaching-based, aerobic and yoga) on depressive symptoms among women during the postpartum period. Similar methods to calculate aggregated effects were used, namely SMD using a random effects model. All the included meta-analyses weighted the studies to give larger samples more influence. As can be seen, a SMD reduction in depression symptoms was observed for all the meta-analyses, favouring the exercise interventions group. Carter et al. [30] found a medium, significant SMD, although the I^2^ was 85%. The results of a post hoc sensitivity analysis presented small, significant results and a moderate heterogeneity (SMD = −0.25, 95% CI: −0.39 to −0.11, *p* < 0.001, I^2^ = 29%). Daley et al. [31] found a large, significant SMD. However, a significant heterogeneity between studies was found when one trial (included exercise as a cointervention with social support) was eliminated. The SMD was reduced to a small effect size, but the heterogeneity was zero. McCurdy et al. [32], Pritchett et al. [33] and Poyatos-Leon et al. [34] found small, significant SMD. The heterogeneity was 37% in the McCurdy study [32], 85% in the Pritchett study [33] and 33% in the Poyatos-Leon study [34].

### 3.6. Subgroup Analyses

The study of Carter et al. [30] performed four subgroup analyses. First, this study found that targeted prevention or treatment interventions yielded a greater effect size (SMD = −0.75, 95% CI: −1.22 to −0.28, *p* = 0.002) compared to the universal prevention interventions (SMD = −0.52, 95% CI: −0.99 to −0.05, *p* = 0.030). Second, it showed that interventions with active exercise-oriented components yielded larger effects (SMD = −1.19, 95% CI: −1.84 to −0.53, *p* <0.001) than those with coaching/motivational components (SMD = −0.21, 95% CI: −0.37 to −0.05, *p* = 0.009. Third, it showed that, when tested against an active control, the exercise-based interventions yielded a smaller effect (SMD = −0.46, 95% CI: −0.86 to −0.05, *p* = 0.03) than those tested against nonactive control groups (SMD = −0.70, 95% CI: −1.09 to −0.32, *p* < 0.001). Fourth, interventions with a shorter duration (SMD = −1.72, 95% CI; −3.05 to −0.39, *p* = 0.010) yielded larger effect sizes than those of longer durations (SMD = −0.52, 95% CI: −0.84 to −0.19, *p* = 0.002).

The study of Daley et al. [31] excluded one trial that included exercise as a cointervention with social support. The SMD was reduced to −0.42 (95% CI: −0.90 to 0.05). The weighted mean difference in the size of the effect of the Edinburgh Postnatal Depression Score was −2.03 (95% CI: −4.34 to 0.29).

The study of McCurdy et al. [32] conducted sub-analyses and showed no difference between supervised and unsupervised exercises. Although analyses with only women with postpartum depression, before starting the intervention (10 trials), exercise had a moderate effect in treating depressive symptoms (SMD = 20.48, 95% CI: 20.22–20.73, I^2^ = 42%). Comparing supervised and unsupervised exercises, only supervised exercise improved depressive symptoms (SMD = 20.74, 95% CI: 20.40–21.07, I^2^ = 27%).

In the study of Poyatos-Leon et al. [34], the PPD status subgroup analysis revealed an effect size of 0.67 (95% CI: 0.44–0.90) for mothers with PPD and 0.29 (95% CI: 0.14–0.45) for mothers without PPD. In the study of Pritchett et al. [33], no differences were observed comparing “depressed” postpartum populations (SMD = −0.32, 95% CI: −0.63 to −0.00, I^2^ = 55%) and general postpartum populations (SMD = −0.57, 95% CI: −1.12 to −0.02, I^2^ = 92). Additionally, no differences were observed comparing exercise-only interventions (SMD = −0.56, 95% CI −1.13 to 0.01, I^2^ = 89%) and exercise with cointerventions (−0.35, 95% CI = −0.66 to −0.04, I^2^ = 72%). In addition, no differences were observed comparing group exercise interventions (SMD = −1.10, 95% CI: −1.99 to −0.21, I^2^ = 93%) and participant choice interventions such as exercise counselling with a personal choice of exercise (often exercising alone) (SMD = −0.20, 95% CI: −0.33 to −0.06, I^2^ = 0%).

### 3.7. Pooled Summary SMD across Meta-Analyses

**Figure 2:**
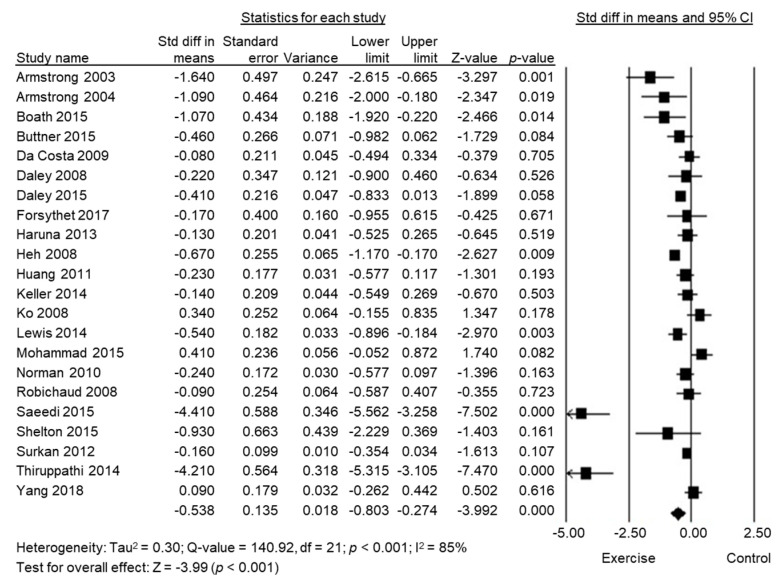
Forest plot of the standard mean differences of exercise intervention on postpartum depressive symptoms by the study.

It was possible to pool the SMD from all the meta-analyses (*n* = 5), with 2564 women in the exercise group and 2626 in the control group, from 63 trials, displayed in Figure 2. This established that exercise had a significant, small effect on depressive symptoms among women during the postpartum period (SMD = −0.41, 95% CI −0.50 to −0.32, *p*< 0.001) using fixed models. There was a low heterogeneity (I^2^ = 2%).

### 3.8. Pooled Summary SMD across Studies without Overlap

**Figure 3:**
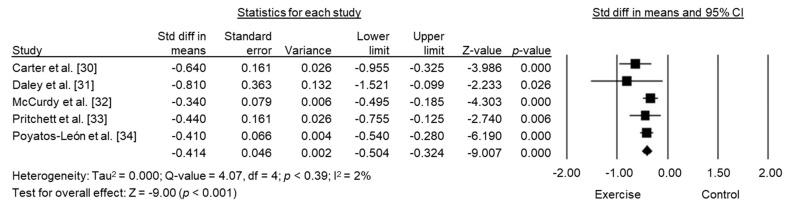
Forest plot of the standard mean differences of exercise intervention on postpartum depressive symptoms by the included meta-analyses.

The overlap of single studies within the five included meta-analyses caused a reduction in the final number of studies to 22 original studies. This analysis revealed that exercise had a significant, moderate effect on depressive symptoms among women in the postpartum period (SD = −0.53, 95% CI −0.80 to −0.27, *p* < 0.001, I^2^ = 85.9) using a random-effects model (Figure 3).

## 4. Discussion

The purpose of this study was to conduct a systematic review of previous meta-analyses addressing the effects of exercise on PPD symptoms. This systematic review included five meta-analyses that comprised 6141 participants. Excluding the overlapping studies, the sample was reduced to a total of 2419 participants. We found that exercise significantly reduces PPD symptoms, with a small effect. We processed the analyses with only studies that were not overlapping, and the results remained the same: exercise significantly reduces PPD symptoms, with a moderate magnitude. Compared with traditional approaches that present a pooled effect size lower than 0.3 for any depression-related outcomes [36], exercise seems to be a feasible alternative. In addition, our results can be compared with a meta-analysis that found a significant small effect of low-intensity psychological interventions (e.g., as online cognitive behavioural therapy and self-help books) versus the usual care for depression in the general population [37]. Exercise has recognised benefits for women during the postpartum period (e.g., weight loss and pelvic floor strengthening) [38,39,40].

Moreover, no detrimental effects, except temporary changes in the composition of breast milk following maximal exercise [41], were found. In this sense, we might recommend exercise as a feasible alternative to control PPD symptoms. Since no serious adverse events were reported, exercise might be considered a safe intervention for this target population. However, we would like to highlight the need for more RCTs exploring the physiological and medical (after) effects of exercise in women with PPD symptoms. For example, the dose–response of exercise remains unknown so far.

Comparing the results of the included meta-analyses, the study of Daley et al. [31] presented the largest effect of exercise on PPD symptoms. However, this result considered one trial that included exercise as a cointervention. In addition, the AMSTAR quality of that particular meta-analysis was low, mainly because they did not account for the possible risk of bias in the individual studies. In second place, the study of Carter et al. [30] found a significant, moderate effect of exercise on PPD symptoms. However, when a sensitivity analysis was conducted, eliminating the studies with a high risk of bias, the magnitude of the effect became small. A small effect of exercise on PPD symptoms was found in the studies from McCurdy et al. [32], Pritchett et al. [33] and Poyatos-Leon et al. [34]. The McCurdy study’s subgroup analyses showed that, for women with depression, exercise improved the odds of resolving depression post-intervention by 54%. However, we must consider that the McCurdy meta-analysis was classified as low-quality, mainly because it did not account for possible biases in the individual studies.

One point that must be highlighted is that the exercise was not tested as an exclusive treatment. PPD is a serious problem that can put both the mother and the baby at risk. In most cases, medication is used as the first option or a combination of treatments such as counselling or therapies [42]. Thus, many times, exercise appears as an adjunctive treatment. In Daley’s meta-analysis [31], three out of five trials reported that the participants received other standard treatments. In Pritchett´s meta-analysis [33], five included studies presented cointerventions, such as dietary, an educational section on postpartum issues or social support. In the Carter [30], McCurdy [32] and Poyatos-Leon [34] meta-analyses, no information about adjunctive treatments was available.

We speculate that the source of the large heterogeneity between the studies came from interventions and outcomes. A variable range of exercise types was used in the trials included in the meta-analyses. The different measures and classifications were used to classify women with PDD.

## 5. Conclusions

As practical implications, this study provided a synthesis of reviews that practitioners and policymakers can use as an evidence map of the effectiveness of exercise on PPD symptoms. Only one study reported the level of evidence, and it was low. For future research, it is important to evaluate the preventive role of exercise during gestation on PPD symptoms, evaluate the dose–response of exercise and clarify the effects of different intervention modalities (e.g., frequency, intensity, time and type—FITT principles). Additionally, future studies can explore meta-regression analyses.

The strength of our study is that we conducted a comprehensive search including only the highest level of evidence (meta-analyses of RCT). Moreover, we provided a pooled effect size across the included studies to demonstrate the beneficial effects of exercise on PDD symptoms. Our results are of high interest to clinicians and researchers in the area considering exercise as an effective way to reduce depressive symptoms among postpartum women. However, several limitations should be acknowledged, mostly reflected by limitations in the original studies. There was a limited number of eligible meta-analyses. The meta-analyses varied in their aims and, mostly, in the type of exercise interventions. For future studies, it is important to understand the exercise characteristics (e.g., frequency, intensity, time and type of exercise) that are most effective.

## Figures and Tables

**Table 1:** Summary of the meta-analyses included in the current study following the PICO search strategy.

Reference	Included RCT’s	Population	Interventions Characteristics	Comparison	Outcomes Measures
Carter et al. [30]	17	1927 primiparous or multiparous postnatal women.	Exercise-based (supervised, unsupervised, coaching-based, motivational, behavioural-oriented, universal, targeted or treatment based, in a community or clinical context).	Any control condition (including exercise).	Depression symptoms using a validated assessment tool (e.g., EPDS and PHQ).
Daley et al. [31]	5	221 women who were between 4 weeks and 18 months postpartum.	The exercise was defined as any planned, structured and repetitive bodily movement. Trials involving exercise with additional interventions (co-interventions) were eligible.	Social support intervention and standard care.	Clinical interview screened for probable depression using a recognised or diagnosed according to the clinical judgment of a health professional.
Mc Curdy et al. [32]	16	1327 postpartum women with and without depression.	Postpartum exercise (supervised or unsupervised exercise interventions).	Standard care.	Depressive symptoms or depressive episodes assessed by a validated questionnaire (e.g., EPDS, CES-D and HDRS).
Pritchett et al. [33]	13	1734 women up to 1 year postpartum.	Aerobic exercise, counselling exercise and group exercise.	Standard care.	Depressive symptoms measured by questionnaire or diagnostic interview.
Poyatos-León et al. [34]	12	932 pregnant women with a single foetus and an uncomplicated pregnancy or women who had a child aged between 6 weeks and 18 months: 471 in the intervention group and 461 in the control group.	Stretching and breathing exercises, a walking program, cardiovascular exercises, mixed cardiovascular and strength exercises, Pilates and yoga exercises and home-based programs.	Any intervention during pregnancy and postpartum period.	Depression scale: the EPDS or the BDI.

Abbreviations: BDI, Beck Depression Inventory; CESD-D, Center for Epidemiological Studies Depression Scale; EPDS, Edinburgh Postnatal Depression Score; HDRS, Hamilton Depression Rating Scale; PHQ, Patient Health Questionnaire.

**Table 2:** Quality of the meta-analyses according to the AMSTAR 2 criteria.

AMSTAR 2 Criteria	Carter et al. [30]	Daley et al. [31]	McCurdy et al. [32]	Pritchett et al. [33]	Poyatos-León et al. [34]
1. Did the research questions and inclusion criteria for the review include the components of PICO?	V	-	-	V	-
2. Did the report of the review contain an explicit statement that the review methods were established before the conduct of the review, and did the report justify any significant deviations from the protocol?	V	-	-	V	V
3. Did the review authors explain their selection of the study designs for inclusion in the review?	V	V	V	V	V
4. Did the review authors use a comprehensive literature search strategy?	V	V	V	V	V
5. Did the review authors perform study selection in duplicate?	V	X	V	V	V
6. Did the review authors perform data extraction in duplicate?	V	V	V	V	V
7. Did the review authors provide a list of excluded studies and justify the exclusions?	V	V	V	V	V
8. Did the review authors describe the included studies in adequate detail?	V	V	V	V	V
9. Did the review authors use a satisfactory technique for assessing the risk of bias (RoB) in individual studies included in the review?	V	V	V	V	V
10. Did the review authors report on funding sources for the studies included in the review?	-	-	-	-	-
11. If meta-analysis was performed, did the review authors use appropriate methods for statistical combination of results?	V	V	V	V	V
12. If meta-analysis was performed, did the review authors assess the potential impact of RoB in individual studies on the results of the meta-analysis or other evidence synthesis?	V	-	V	V	V
13. Did the review authors account for RoB in individual studies when interpreting/discussing the results of the review?	V	-	-	V	V
14. Did the review authors provide a satisfactory explanation for, and discussion of, any heterogeneity observed in the results of the review?	V	V	V	V	V
15. If they performed quantitative synthesis, did the review authors carry out an adequate investigation of publication bias (small study bias) and discuss its likely impact on the review results?	V	-	-	-	V
16. Did the review authors report any potential sources of conflict of interest, including any funding they received for conducting the review?	V	V	V	V	V
	MQR	LQR	LQR	MQR	MQR

Abbreviation: MQR, moderate quality review; LQR, low-quality review; V, meets the criteria; X, does not meet the criteria.

**Table 3:** Review findings.

Reference	SMD (95% CI)	I^2^ (%)	Conclusions
Carter et al. [30]	−0.64 (−0.96 to −0.33)	86.0%	Statistically significant medium treatment effect of exercise over control conditions for depression symptoms in postpartum women up to 52 weeks after childbirth.
Daley et al. [31]	−0.81 (−1.53 to −0.10)	81.7%	Exercise can reduce postpartum depression, but this finding is contingent on one trial that included exercise as a co-intervention.
McCurdy et al. [32]	−0.34 (−0.50 to 0.19)	37%	Post-intervention depressive symptoms were lower in the exercise compared with the control group. In women with depression, exercise improved the odds of resolving depression post-intervention by 54%.
Pritchett et al. [33]	−0.44 (−0.75 to −0.12)	85.0%	Exercise interventions significantly reduced depressive symptoms
Poyatos-León et al. [34]	−0.41 (−0.28 to −0.54)	33.1%	Decrease in postpartum depressive symptom scores favour of the physical activity group.

Abbreviations: I^2^, I-squared statistic for heterogeneity; SMD, standardised mean difference.

## Data Availability

Please contact the corresponding author to discuss the availability of the data and materials.
